# Understanding the Effects of Metabolites on the Gut Microbiome and Severe Acute Pancreatitis

**DOI:** 10.1155/2021/1516855

**Published:** 2021-10-19

**Authors:** Shijie Ye, Chenli Si, Jie Deng, Xiaohu Chen, Lingming Kong, Xiang Zhou, Weiming Wang

**Affiliations:** ^1^Wenzhou Medical University, Wenzhou, China; ^2^Key Laboratory of Diagnosis and Treatment of Severe Hepato-Pancreatic Diseases of Zhejiang Province, The First Affiliated Hospital, Wenzhou Medical University, Wenzhou, China; ^3^Department of Pathology, The First Affiliated Hospital, Wenzhou Medical University, Wenzhou, China; ^4^Department of Breast Surgery, The First Affiliated Hospital of Wenzhou Medical University, Wenzhou, Zhejiang, China; ^5^Department of Hepatopancreatobiliary Surgery, The First Affiliated Hospital of Wenzhou Medical University, Wenzhou, Zhejiang, China

## Abstract

Acute pancreatitis (AP) is an inflammatory disease of the pancreas. The severity is classified as mild (MAP), moderately severe (MSAP), or severe (SAP). In patients with SAP, organ dysfunction can occur in the early stage of the disease course, accompanied by secondary infection, with a mortality rate of 36%–50%. In the late stage SAP, infection-related complications caused by pancreatic necrotic tissue and peripancreatic effusion are the main causes of death in patients. Dysbacteriosis of intestinal microflora, barrier dysfunction of intestinal mucosa, and translocation of enteric bacteria are considered to be the main causes of infection of pancreatic necrotic tissue and peripancreatic effusion. During the past few years, increasing attention has been paid to the metabolic activities of intestinal microflora in SAP, which plays an important role in the metabolic activities of the human body. This review is aimed at bringing together the most recent findings and advances regarding the gut microbial community and associated gut microbial community metabolites and illustrating the role of these metabolites in disease progression in severe acute pancreatitis. We hope that this review will provide new ideas and schemes for the treatment of SAP in the clinical settings.

## 1. Introduction

The incidence of acute pancreatitis is increasing worldwide, placing a burden on health services [1]. Acute pancreatitis (AP) is a potentially lethal inflammatory disorder of the pancreas. The initiation site of AP is the damaged pancreatic acinar cells, and eventually, trypsin activation leads to pancreatic autodigestion [2]. The activation of nuclear factor kappa B (NF-*κ*B) plays an important role in the pathogenesis of acute pancreatitis [3]. Some studies have shown that pancreatic stellate cells might also have a key early role [4].

The revised Atlanta classification of AP identified two phases of the disease: early and late, while severity is classified as mild, moderately severe, and severe [5]. Severe AP (SAP) accounts for 5%–10% of the cases, accompanied by organ failure (≥48 h). SAP has a high mortality rate in the early stage and a higher mortality rate in the late stage of infection. Pancreatic necrosis occurs in most patients with persistent organ failure, with a mortality rate of more than 30% [6]. Patients with SAP often suffer from intestinal perfusion in the early stage of onset, and active fluid resuscitation causes intestinal ischemia-reperfusion injury. The appearance of intestinal ischemia-reperfusion is associated with the alteration of intestinal microflora and the impairment of intestinal barrier function [7]. Interestingly, patients with SAP are often accompanied with significant changes in the intestinal microflora, including a decrease in gut microbiota diversity, an increase in *Enterococcus* and Enterobacteriaceae, and a decrease in *Bifidobacterium* [8]. The intestinal microflora dysbiosis can promote bacterial translocation (BT) by inducing the mucosal immune dysfunction and increasing the gut permeability [9]. In recent years, a number of studies suggest that the intestinal microflora play a key role in the pathological course of AP. Although previous studies have reported potential routes and mechanisms, these results are not well understood, and more information is required from future studies [10]. In this review, the basic situation and the translocation of intestinal microflora in SAP were evaluated, and several important intestinal bacteria and their metabolites that are linked with SAP were discussed.

## 2. Etiology and Pathophysiology of Severe Acute Pancreatitis

### 2.1. Etiology

Although there are many etiologies of SAP, its pathogenesis remains controversial. The risk of SAP is increased by several factors, including genetic, environmental, and organismal metabolic factors [11]. The two major etiologies of AP, gallstone and alcoholic AP, which usually account for approximately 60% to 80% of all cases, differ in different countries and regions. Gallstones occur in most developed countries; 45% of cases of AP are caused, and 20% are due to alcohol abuse [12, 13]. Some researchers have pointed out that the coronavirus disease 2019 may also be regarded as a new cause of AP, but it requires further investigation [14].

Hypertriglyceridemia is the third most common cause of AP. According to previous studies, the prevalence of hypertriglyceridemia-induced pancreatitis (HTGP) is approximately 22% and accounts for 5% of all AP cases. In women, HTGP accounts for approximately 56% of all AP cases during pregnancy [15]. Other uncommon causes include medical therapy, endoscopic retrograde cholangiopancreatography, hypercalcemia, infection, genetics, autoimmune diseases, and surgical trauma [16].

### 2.2. Cellular Mechanisms

Pathological calcium signaling, mitochondrial dysfunction, premature trypsinogen activation within acinar cells and macrophages, endoplasmic reticulum stress, impaired unfolded protein response impaired autophagy, and impaired autophagy are common to the core pathogenesis of AP [17]. A recent study identified metabolites produced by the gut microbiota that inhibit hypoxia inducible factor (HIF-2 *α*) through a high-throughput microbial metabolite screening. This transcription factor plays an important role in the physiology of intestinal iron absorption by increasing the iron storage protein ferritin, which leads to a decreased intestinal iron absorption by the host. This study also demonstrates a novel mechanism of metabolic crosstalk between metabolites of the gut microbiome and the host intestinal epithelium, which regulates intestinal and systemic iron homeostasis [18]. Ferroptosis is a reactive oxygen species-dependent, regulatable form of cell death that is morphologically, biochemically, and genetically distinct from other forms of cell death, such as apoptosis, necrosis, and autophagy. Ferroptosis is associated with two main biochemical features, namely, iron accumulation and lipid peroxidation [19, 20]. A recent study showed that utilized two gut microbial metabolites, reuterin and 1,3-diaminopropane, can inhibit intestinal HIF-2 *α* activity *in vitro* and *in vivo* by preventing dimerization of HIF-2 *α* with aryl hydrocarbon receptor nuclear translocator, ultimately preventing tissue iron accumulation in a mouse model of iron overload [18]. Through these experimental findings, we speculated that disturbance in the intestinal microflora would lead to disruption of body iron homeostasis. Moreover, changes in intestinal microflora metabolites may promote the translocation of gut flora in patients with AP by inducing iron death of intestinal epithelial cells (IECs), leading to the development of SAP.

## 3. Summary of Microbial Composition Diversity of the Human Gut Microbiome

Changes in the composition of the gut microflora are associated with host physiology and pathology. An increasing number of studies have defined microbial-host interactions at the molecular level. Moreover, the intestinal flora and metabolites are closely related to the host [21].

Approximately 10–100 trillion microbes live in the human body. The human intestine contains a complex and dynamic microbial ecosystem, and the number of intestinal microflora even exceeds the number of human cells [22]. The adult intestinal microflora is mainly composed of members of Bacteroidetes and Firmicutes, accounting for approximately 90% of the adult intestinal microflora [23]. There are great differences in the composition of intestinal microflora between individuals and multiple healthy microbiome states within the healthy host space [24]. The intestinal microflora is thought to be a forgotten organ [25]. The intestinal flora develops during the host's infancy and eventually reaches its adult form. For many years, it was thought that the environment of the fetus in the uterus was sterile, and infant intestinal colonization begins at delivery. However, this dogma of a sterile *in utero* environment has been challenged. A growing body of scientific evidence has indicated the presence of bacteria in the placenta [26], umbilical cord [27], and amniotic fluid [28]. In healthy full-term pregnancies, intestinal microbial communities of infants are influenced by birth patterns and nutritional factors. The intestinal microflora reaches a stable level by the age of three years [29]. Many environmental parameters, including intestinal pH, oxygen level/redox status, nutrients, water, and temperature, are related to the composition of intestinal microflora. These parameters allow various populations to perform different activities while interacting with their living environment and enable the intestinal microflora to better adapt to the environment of the human host [30].

## 4. Bacterial Translocation from Gut

Bacterial translocation is defined as the process by which the gut bacteria and/or their products spread through the intestinal mucosa into the normal sterile extraintestinal sites [31]. Pancreatic necrosis is a serious local complication in the acute pancreas, and it is often closely associated with pancreatic infection.

Gut-derived bacteria can infect the diffused or local area of nonviable parenchyma, which is initially sterile [32]. Several studies detected intestinal microflora translocation in the blood of patients with SAP using 16S rDNA sequencing [33, 34]. In patients with AP, opportunistic pathogens in the gut, including *Escherichia coli*, *Shigella flexneri*, *Enterobacteriaceae bacterium*, *Acinetobacter lwoffii*, *Bacillus coagulans*, and *Enterococcus faecium*, are predominant during bacterial translocation [34].

The intestinal barrier system consists of intestinal epithelial cell junction complex and its secretions, intestinal-associated immune cells, and intestinal normal flora and includes the mucosal chemical barrier, mechanical barrier, immune barrier, and biological barrier, with the last one also known as gut microbiota. Microbial dysbiosis may occur during the onset of acute pancreatitis [35]. During the development of SAP, the intestinal permeability increased, and the expression level of diamine oxidase in the serum of patients with SAP was significantly higher. This is due to the severe damage in the intestinal mucosa during SAP and the transient increase caused by the release of a large amount of diamine oxidase activity (DAO) into the blood [36]. A study has shown that intestinal microflora dysbiosis occurred in patients with AP, and it is associated with a decrease in probiotics and an excessive proliferation of opportunistic pathogenic bacteria. At the same time, the study also found that intestinal microflora imbalance can cause a decrease of short-chain fatty acid- (SCFA-) producing bacteria, which in turn affects the integrity of the intestinal barrier and then worsens the severity of AP [37]. In addition, the production of a large number of inflammatory factors also plays an important role in the translocation of intestinal microflora. In patients with SAP, inflammatory factors are released in large quantities, leading to ischemia-reperfusion injury of the intestinal mucosa, which induces severe oxidative stress in the intestinal mucosa and activation of caspase-3. These pathological changes aggravate apoptosis in the intestinal mucosa cells. The tumor necrosis factor-alpha (TNF-*α*) is also important in gut mucosal injury [38]. In the early stage of SAP in rats, intestinal immunosuppression may lead to bacterial and endotoxin translocation. The immune barrier was compromised, and secretory immunoglobulin A levels decrease. These changes reduced the ability of the gut to resist colonization [10]. The diversity of intestinal microflora can stimulate the production of different IgA, since most of the commensal microflora are encapsulated by IgA [39, 40]. IgA can alter bacterial metabolism, eliminate mucosal inflammation, and maintain immune homeostasis [41]. A recent study identified that intestinal microbiota and NLRP3 (NOD-, LRR- and pyrin domain-containing 3) interact in the process of AP [42]. Patients with SAP die as a result of early multiple organ failure (MOF) and later development of infectious complications [43]. Proinflammatory responses lead to systemic inflammatory response syndrome. Further development of AP may lead to early MOF. Gut-derived infection can also exacerbate the condition [44]. Many pieces of evidence have confirmed that intestinal failure plays an important role in disease development, and translocation of intestinal flora can lead to secondary infections, including infectious pancreatic necrosis [45].

## 5. Intestinal Microflora and AP

With the change in lifestyle and dietary habits in modern society and the increased exposure to environmental risk factors, the incidence of many diseases, including AP, has increased dramatically. According to the different complication in patients, AP can be divided into mild AP (MAP) with edema as the main manifestation and SAP with hemorrhage and necrosis as the main manifestation [5]. Patients with MAP have a low mortality rate and generally have no organ failure and complications. Meanwhile, patients with SAP are prone to organ dysfunction in the early stage of the disease, accompanied by secondary infection; most patients with persistent organ failure have pancreatic necrosis and mortality rate of at least 30% [6]. If infection occurs later, the mortality rate is higher.

At present, theories for the pathogenesis of AP mainly include pancreatic enzyme autodigestion, inflammatory response, apoptosis, and microcirculation changes [46]. In addition, the theory of intestinal bacterial translocation has received increasing attention in recent years.

Patients with early AP are accompanied by disturbance of the intestinal microflora, which leads to intestinal barrier disorder. Studies have shown that bacterial translocation occurs within 1–2 weeks of AP occurrence, which often leads to intestinal-derived infection [44]. The emergence of new intestinal microflora detection methods has led to an increasing number of studies exploring the specific changes in the abundance and diversity of intestinal flora in the course of AP.

The role of probiotics in AP remains controversial; therefore, studies have reassessed the metabolic effects of probiotics on AP. The mortality rate in the probiotic treatment group was higher than that in the placebo group, which may be related to the disturbance of intestinal flora and intestinal barrier or the metabolic changes of intestinal flora [47]. In addition, double-blind experimental studies have shown that prophylactic administration of probiotics increased the susceptibility rate of AP [48]. These results suggest that the role of probiotics in AP should be elucidated.

## 6. Intestinal Microflora Metabolites

The intestinal microflora has a high metabolic activity (Table 1), which can transform the source of host and dietary components into different metabolites. Some metabolites are beneficial, and some are harmful [49]. The metabolites that are beneficial to the host include lactic acid, bile acids (BAs), SCFAs, and bacteriocins, which are generally considered antibacterial factors and play a key role in the prevention of pathogenic infection.

### 6.1. Short-Chain Fatty Acids

SCFAs are the main metabolic products of saccharolytic fermentation of nondigestible carbohydrates by the intestinal microflora [61]. The main SCFAs produced in the human intestine are formate, acetate, propionate, and butyrate. SCFAs can be absorbed by the intestinal mucosa and act as an energy source. In addition, SCFAs can regulate gene expression as a signaling molecule [61]. Butyrate is mainly produced by *Faecalibacterium prausnitzii*, *Eubacterium rectale*, *Eubacterium hallii*, and *Ruminococcus bromii* [62]. SCFAs regulate host biological responses through two key pathways. The first mechanism involves the regulation of gene expression by inhibiting histone deacetylase (HDAC) activity. Butyrate and propionate are considered the main intrinsic HDAC inhibitors. The second mechanism involves signaling through G-protein-coupled receptors (GPRs). The major G-protein-coupled receptors activated by SCFAs are GPR41, GPR43, and GPR109A [63]. Several studies have uncovered new mechanisms involved in SCFA regulation of immune cell development and suppression of inflammation [64].

SCFAs exert anti-inflammatory effects in both innate and adaptive immunity by inhibition of HDACs and GPCRs present in IECs and immune cells [65]. A study has shown that activation of GPR109A promotes the activation of colonic macrophages and dendritic cells and eventually induces IL-10-producing T cells. In addition, GPR109A plays an important role in butyrate-mediated induction of IL-18 production in colon epithelial cells [66]. Moreover, SCFAs can activate the NLRP3 inflammasome [50]. Recently, a study demonstrated that SCFAs promoted the AMPs RegIII*γ* and *β*-defensins in a GPR43-mediated signaling pathway [67]. SCFAs also play a role in the anti-inflammatory effects partly through HDAC inhibition [68]. Butyrate regulates the transcription of certain cytokine genes, including IFN-*γ*, TNF-*α*, and NF-*κ*B, by inhibiting HDAC activation. [69].

SCFAs, particularly butyrate, play a positive role in intestinal epithelial cells and overall intestinal health [70]. In addition, SCFAs have effects on cell proliferation and immune response [71]. Butyrate is the main energy metabolic substrate of colonocytes [72]. A study demonstrated that butyrate-producing bacteria increased colonization capacity in mucus- and lumen-associated microbiota in Crohn's disease and improved the epithelial barrier integrity *in vitro* in a disrupted microbial community [70]. Of the SCFA produced in the colon, butyrate is the main regulator of tight junction protein (TJP), and it has been shown to enhance intestinal barrier function through increased expression of claudin 1 and Zonula Occludens-1 (ZO-1) and occludin redistribution [73]. Meanwhile, translocation of bacteria and cell wall components may be associated with increased intestinal barrier permeability [74]. *In vitro*, the addition of butyrate to monolayer Caco-2 cells can promote the assembly of ZO-1 and occludin, which depends on the activation of AMP-activated protein kinase without changing their expression levels [75]. Butyrate and propionate could increase intestinal mucin MUC2 expression by the induction of MUC2 mRNA expression in human goblet-like cell line LS174T [76].

### 6.2. Bile Acids

BAs are produced in the liver from cholesterol and are further metabolized in the intestine by the gut microbiota [53]. A previous study demonstrated that microbiota regulates BA synthesis and metabolism through farnesoid X receptor (FXR) [77]. The activation of FXR can mediate NF-*κ*B inhibition *in vitro* [78]. Pregnane X receptor (PXR) and vitamin D receptor can be activated by specific BAs [53]. BAs can also function in signaling by activation of the G-protein-coupled receptor TGR5, another nuclear receptor [54]. Hepatocytes directly synthesize BAs, which are amphipathic molecules from cholesterol. This amphipathic structure contributes to the absorption of fat-soluble vitamins and fat emulsification. In terms of immunity, BAs have antimicrobial functions. They are usually secreted in a conjugated manner, helping to increase the solubility of BAs in body fluids [79].

Intestinal microflora can promote BA deconjugation through bile salt hydrolase. This can decrease the solubility of BAs, reduce toxicity, or obtain taurine or glycine [80]. The deconjugated primary BAs can be further converted to secondary BAs by 7*α*-dehydroxylation. A number of bacteria, mostly Clostridia, have this ability [80]. A study showed that bacterial species conferred protection against *C. difficile* infection in antibiotic-treated mice and humans and identified *Clostridium scindens* as a good predictor of resistance [81]. Additionally, chenodeoxycholic acid, a primary BA, can activate innate immunity in the intestine through FXR [82]. A recent study showed that dietary and intestinal microflora can affect the composition of the gut BA pool and modulate an important population of colonic FOXP3+ regulatory T (Treg) cells expressing the transcription factor ROR*γ* [83].

Secondary BAs also play a role in immunity. M1 macrophages are proinflammatory, while M2 macrophages are anti-inflammatory. They are both regulated by the BA-dependent activation of TGR5 [84]. A study has shown secondary BAs protect against intestinal inflammation by inhibiting of epithelial apoptosis and decreasing proinflammatory cytokine levels [85]. In addition, the expression of toll-like receptor 4-NF-*κ*B pathway molecules was significantly inhibited by the activation of TGR5 [86].

### 6.3. Polyamines (PAs)

PAs are small polycationic molecules with a wide array of physiological functions [87]. Spermine, spermidine, and putrescine are widely distributed in human cells, while putrescine, cadaverine, spermidine, and spermine are mainly found in bacteria. PA synthesis is tightly regulated at the molecular level by a mechanism involving *de novo* biosynthesis, catabolism, and specific transport systems [88]. The presence of amino acid precursors or other intermediates is a prerequisite for PA synthesis [89]. The best-known examples of which are the putrescine-specific uptake system and spermidine-preferential uptake system in *Escherichia coli* [90].

Several studies have shown that PA has an impact on bacterial pathogenesis. For example, *Shigella* spp. [91], which lack the ability to synthesize cadaverine due to mutations and deletions in the gene, alter the pathogenicity. Cadaverine can inhibit the damage of the intestinal mucosa caused by enterotoxins. Spermidine helps to enhance resistance of *Shigella* to oxidative stress and fight phagocytosis in macrophages [89]. The intestinal microflora has the ability to synthesize putrescine, spermine, and spermidine, and they produce PAs in the intestinal tract [55, 92]. Intestinal microflora can use arginine to synthesize putrescine through sequential reactions by different bacteria [93]. Furthermore, PAs are associated with virulence levels and viability of certain bacterial pathogens within the host, including *Helicobacter pylori*, *Salmonella enterica* subsp. *enterica* serovar Typhimurium, *Shigella* spp., *Staphylococcus aureus*, *Streptococcus pneumonia*, and *Vibrio cholera* [89]. PAs are required for the growth of intestinal epithelial cells, and apoptosis of intestinal epithelium is also regulated by PAs [94]. High concentrations of PAs in the human intestine can regulate the intestinal epithelial barrier integrity by controlling the expression of TJPs, including ZO-1, occludin, and E-cadherin [56, 95, 96]. A study in rats with L-ornithine-induced pancreatitis showed that pancreatitis is associated with PA metabolism; however, the specific mechanism still needs further study [97]. PA metabolism modulates systemic and mucosal adaptive immunity. Arginine is an important modulator of the immunometabolism of macrophages and T cells that affect their effector functions [98]. Previous studies have shown that simultaneous administration of *Bifidobacterium* spp. LKM512 and arginine to mice can increase intestinal PA levels, which are associated with decreased colonic TNF and IL-6 levels. On the other hand, a high intestinal PA level can also suppress inflammation and induce resistance to oxidative stress [92, 99].

### 6.4. Tryptophan Catabolites

Intestinal microflora can break down proteins to form indole compounds, mainly from aromatic amino acids, such as tryptophan. Tryptophan can be metabolized by intestinal microflora, such as lactobacilli in mice, rather than being limited to proteolytic specialists [100]. Tryptophan is degraded to 3-methylindole and other indoles by different gut microorganisms, such as lactobacilli [101]. The products of bacterial metabolism of tryptophan are bioactive indole-3-aldehyde (IAId), indole-3-propionate (IPA), and indole-3-acetic acid (IAA), which can affect intestinal barrier integrity and immune cell activity by activating the aryl hydrocarbon receptor (AhR) and PXR [102, 103]. Several studies have indicated that tryptophan-related metabolites are closely related to intestinal epithelial function and intestinal barrier function [104]. Furthermore, many studies to date have shown that several bacterial species are able to convert tryptophan into indole and indole derivatives [57]. *L. reuteri* is a major producer of these metabolites and can stimulate AhR activity, which inhibits proinflammatory activity [100, 105]. Additionally, a study found the presence of the phenyllactate dehydratase gene cluster in *Peptostreptococcus* spp., including *P. russellii*, *P. anaerobius*, and *P. stomatis*. These species can convert tryptophan into indoleacrylic acid and IPA [106]. Studies have shown that indole secreted by symbiotic *E. coli* can reduce chemotaxis. Furthermore, the indole can inhibit the attachment of pathogens to the epithelial cells by regulating gene expression that is involved in strengthening the intestinal barrier and mucin production [107]. Indole attenuates TNF-*α*-mediated activation of NF-*κ*B and IL-8 secretion and increases the expression of IL-10. Furthermore, it increases the attachment of pathogenic *E. coli* to HCT-8 cells [107]. A recent study showed the relationship between IPA and the intestinal barrier. *Clostridium sporogenes* can convert tryptophan into tryptamine, indolelactic acid (ILA), and IPA. After comparing the colonization in the intestine between wild-type and fldC mutant *C. sporogenes* in germ-free mice, the study found that the permeability of the intestine is concordant with depleted levels of luminal IPA [58]. IA may mediate AHR to promote intestinal barrier functions and mitigate inflammatory responses in mice and promote goblet cell differentiation and mucus production [106]. *Lactobacillus* spp. can regulate IL-22 mucosal homeostasis through the activation of AHR by IAld [100].

In addition, studies have shown that ILA inhibits the polarization of T helper cell 17 (Th17) cells *in vitro* [108]. The metabolic pathway of tryptophan can affect the differentiation of primary CD4+ T helper cells into Treg cells and TH17 cells through AHR. These two cells play important roles in autoimmune and inflammatory diseases [109].

Tryptamine, a metabolite of tryptophan produced by a variety of gut bacteria [110], is a neurotransmitter associated with intestinal motility [111].

## 7. Other Metabolites

The metabolites discussed in the previous sections are well known to the general public. The following lists the relevant intestinal flora and several specific products produced that may have some impact on SAP.

In the human intestine, *Bifidobacterium* and *Lactobacillus* are probiotic organisms that stimulate antitumor properties and immunity [112]. The main products of *Bifidobacterium* metabolism are lactic acid and acetic acid, which reduce the pH value in the intestine, inhibiting the growth of harmful microbes. This mechanism is particularly evident in the cecum and ascending colon [113]. *Lactobacillus* and *Bifidobacterium* can produce exopolysaccharides (EPSs). EPSs, as effector surface macromolecules, participate in the interaction of bacteria with host cells and intestinal microflora [114]. A novel study found that a secreted protein from *Lactobacillus rhamnosus* GG (LGG), HM0539, protects the intestinal barrier by enhancing the expression of intestinal mucin and preventing intestinal barrier impairment caused by lipopolysaccharide or TNF-*α* [59]. Two proteins produced by LGG, p40, and p75 also have been shown to promote IEC homeostasis [115]. *Faecalibacterium prausnitzii* can secrete seven peptides. They all belong to a protein called microbial anti-inflammatory molecule (MAM). The study has shown that MAM inhibits the NF-*κ*B pathway through *in vivo* experiments in NF-*κ*B-luciferase transgenic mice [60]. Urolithin A (UroA) is an important microbial metabolite of polyphenols from berries and pomegranate fruits. It has anti-inflammatory, antioxidant, and antiaging activities in the human body. A study has shown that UroA and its synthetic analogue (UAS03) can upregulate epithelial tight junction proteins by activating the AhR-nuclear factor erythroid 2-related factor 2-dependent pathways, thereby enhancing the intestinal barrier and reducing the occurrence of inflammation [116].

## 8. Conclusions

In conclusion, the study of microbiota host response is noteworthy as a new direction, and much evidence suggests that bacteria play an important role in pancreatic diseases. In recent years, as a newly discovered “organ” in human body, the importance of intestinal flora in human health has been gradually understood. With the deepening of research, people have a clearer understanding of the role of intestinal microflora in the occurrence and development of SAP. However, its specific mechanism still should be explored and verified. In this study, we reviewed the intestinal microflora and related intestinal microflora metabolites that may play a role in SAP. With the increasing number of studies, this field of research is moving forward, and we believe that new therapeutic interventions based on bacterial-related functions, as well as therapeutic approaches, can be generated in the near future. Researchers are continuously and constantly exploring the pathogenesis of intestinal microflora in SAP at the molecular level. This approach provides a new method for the treatment of SAP. Metabolites of intestinal microflora can be further used in clinical studies and dietary interventions for the treatment of SAP.

## Figures and Tables

**Table 1:** The metabolites produced by the intestinal microflora.

Molecule	Species	Effect	Relevant receptors	Reference
Short-chain fatty acids	*Roseburia* spp. *Eubacterium* spp. *Clostridium* spp. *Ruminococcus* spp. *Faecalibacterium prausnitzii* etc.	Activation of GPCRs HDACs ↓ NF-*κ*B inhibition IgA secretion↑ Proinflammatory cytokines↓ Leukocyte recruitment↑ NLRP3 inflammasome↑ Epithelial barrier integrity↑	GPR43 GPR109A GPR41	[50–52]
Secondary bile acids	*Bifidobacterium* spp. *Bacteroides* spp. *Clostridium* spp. *Eubacterium* spp. *Ruminococcus gnavus* *Peptostreptococcus productus* *Pseudomonas testosteroni* *Lactobacillus plantarum*	Epithelial barrier integrity↑ The expression of toll-like receptor 4-NF-*κ*B pathway↓ Proinflammatory cytokines↓ Cell apoptosis↓	TGR5 FXR PXR VDR	[53, 54]
Polyamines	*Bacteroides fragilis* *Shigella flexneri* *Streptococcus pneumoniae*	Epithelial barrier integrity↑ Expression of tight junction proteins↑ TNF and IL-6 levels↓ Modulates mucosal adaptive immunity	-	[55, 56]
Tryptophan catabolites: 3-methylindole Indole Indole-3-propionic acid (IPA) Indoleacrylic acid (IA) IAId IAA ILA	*Clostridium* spp. *Bacteroides* spp. *Bifidobacterium* spp. *Peptostreptococcus* spp. *Lactobacillus* spp. *Eubacterium* spp. *Escherichia* coli	Epithelial barrier integrity↑ Activation of AHR NF-*κ*B inhibition IL-8 secretion↑ The expression of IL-10↑ Immune cell function↑	AHR PXR	[57, 58]
HM0539	*Lactobacillus rhamnosus*	LPS- or TNF-*α*-mediated barrier injury↓	-	[59]
p75 and p40	*Lactobacillus* sp.	Cell apoptosis↓	-	[60]
MAM	*Faecalibacterium prausnitzii*	NF-*κ*B pathways↓ Th1 and Th2 responses↓	-	[60]
